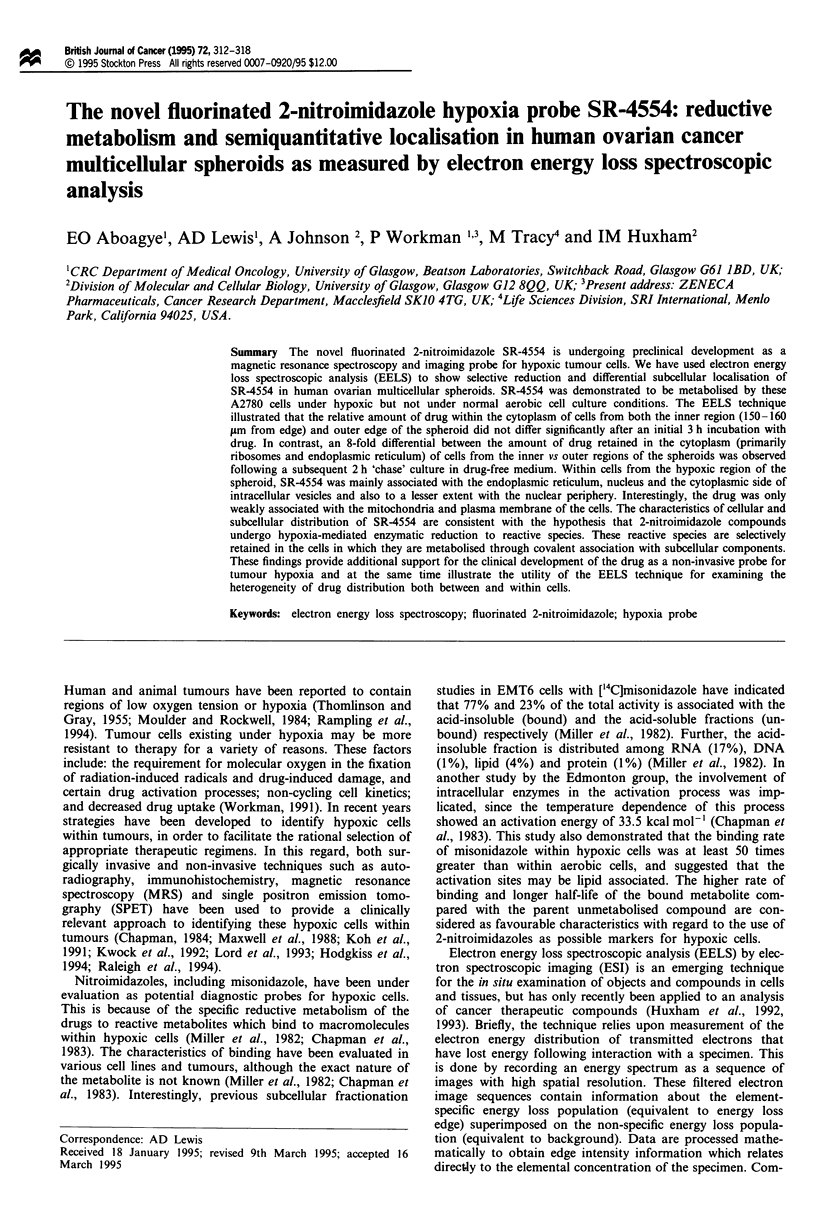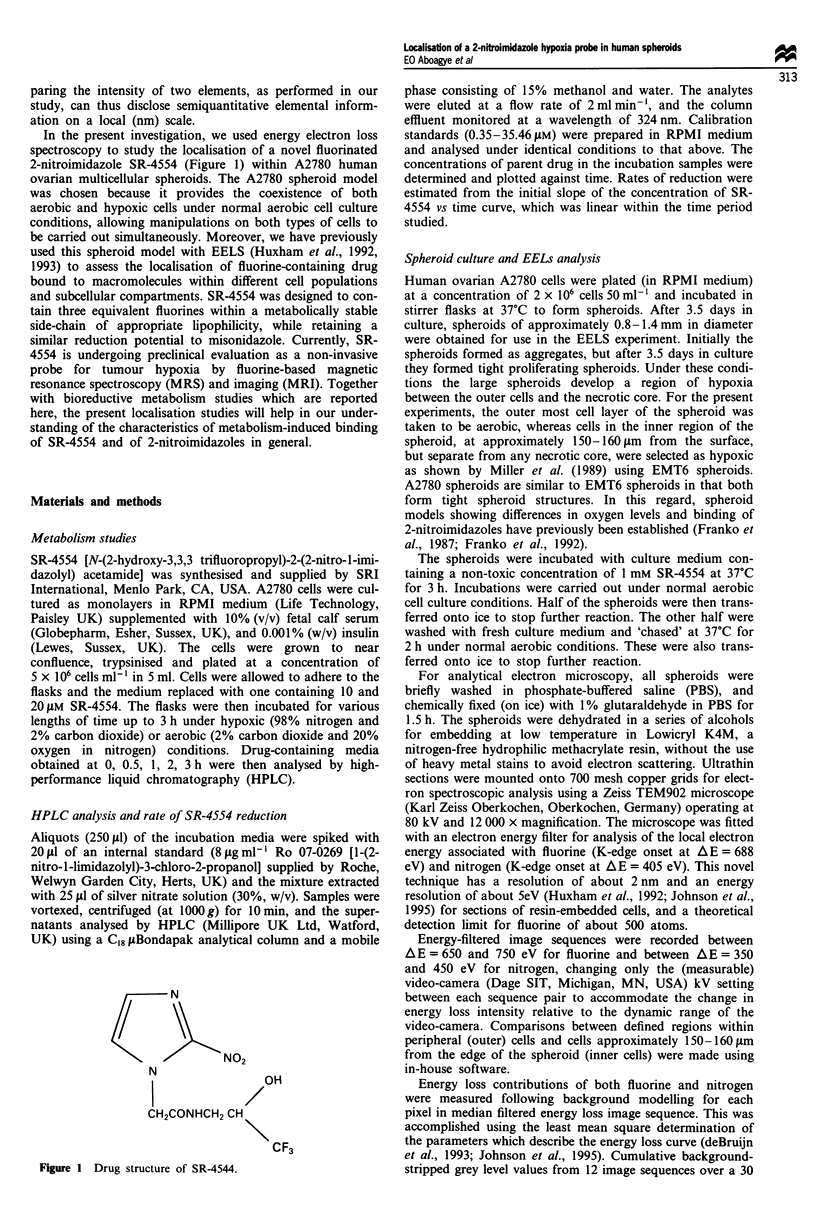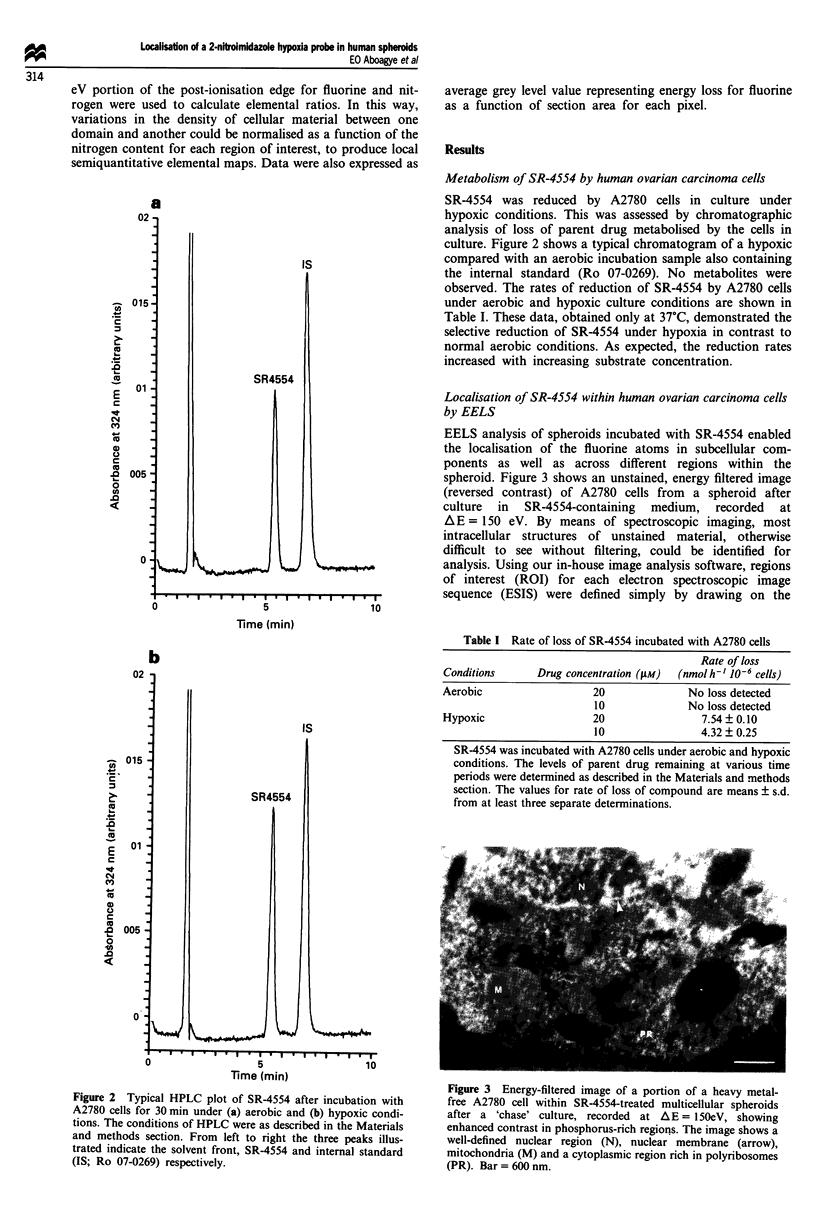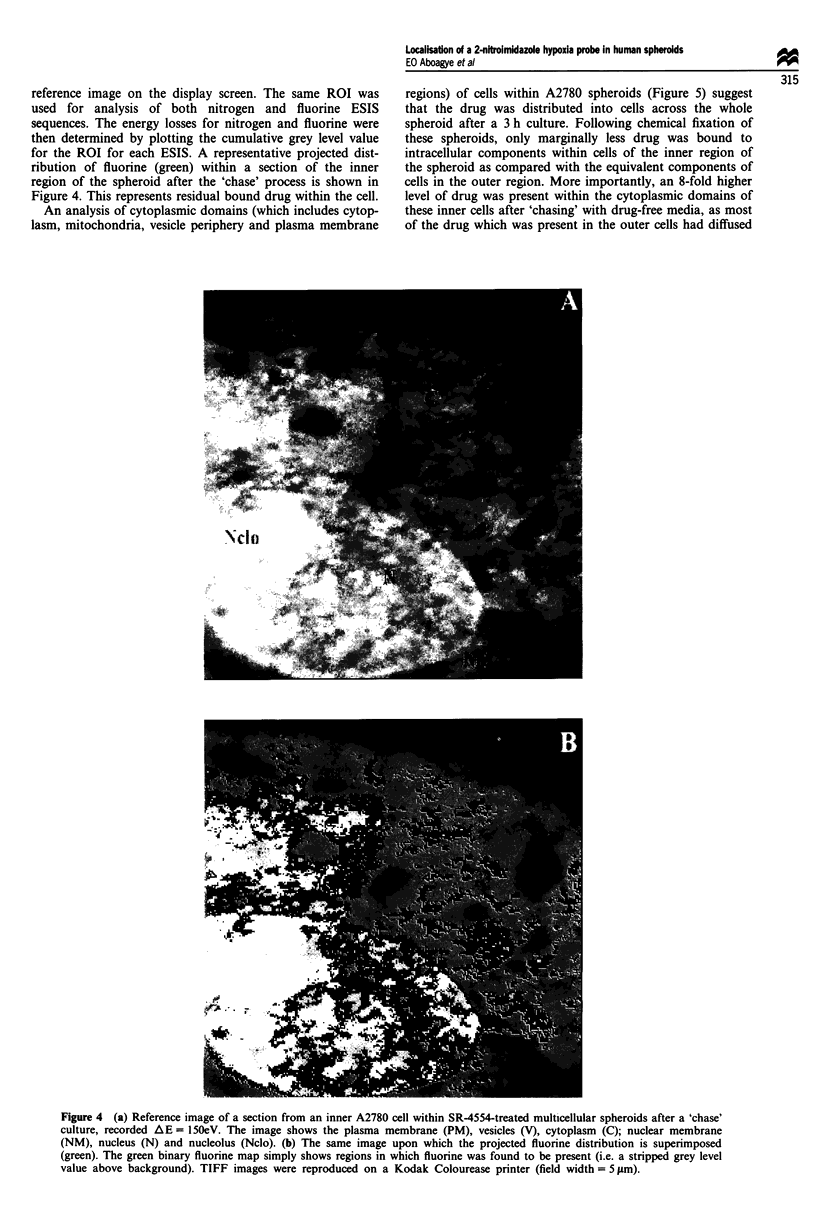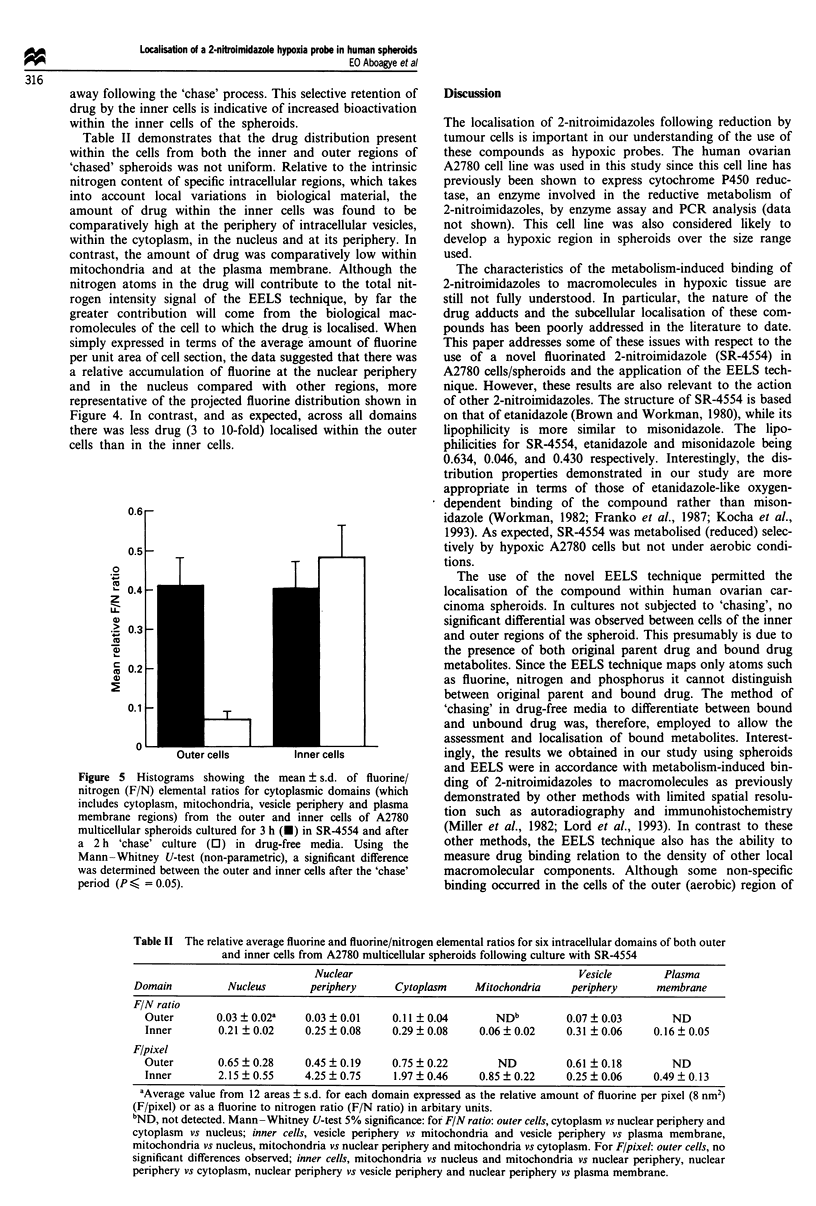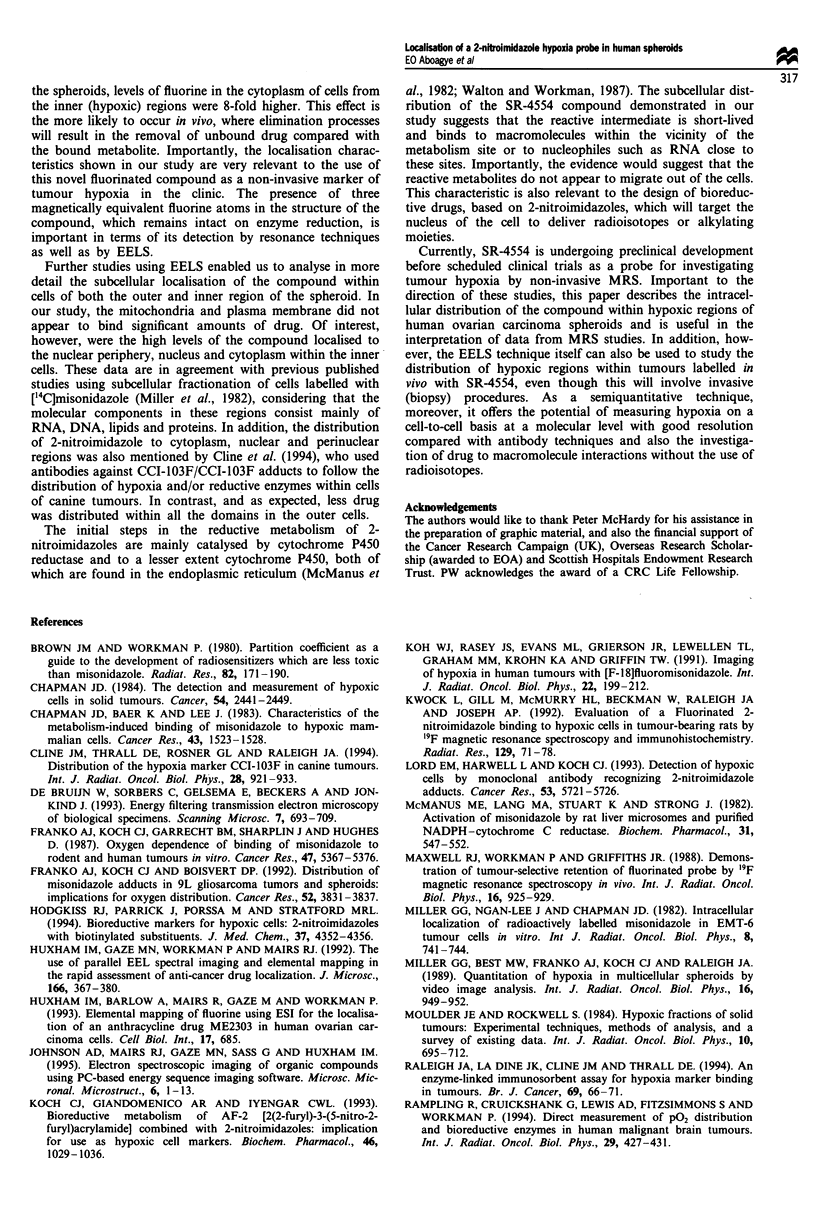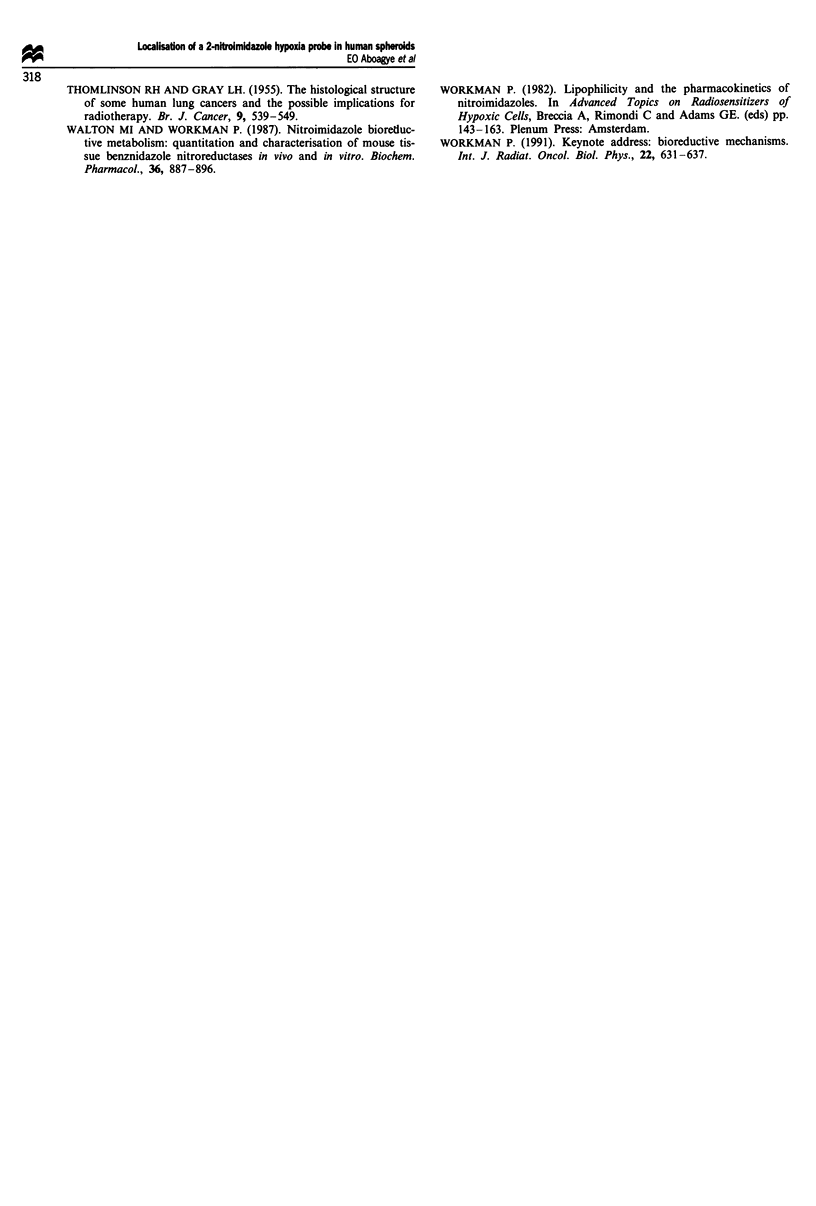# The novel fluorinated 2-nitroimidazole hypoxia probe SR-4554: reductive metabolism and semiquantitative localisation in human ovarian cancer multicellular spheroids as measured by electron energy loss spectroscopic analysis.

**DOI:** 10.1038/bjc.1995.330

**Published:** 1995-08

**Authors:** E. O. Aboagye, A. D. Lewis, A. Johnson, P. Workman, M. Tracy, I. M. Huxham

**Affiliations:** CRC Department of Medical Oncology, University of Glasgow, Beatson Laboratories, UK.

## Abstract

**Images:**


British Journal of Cancer (1995) 72, 312-318
© 1995 Stockton Press All rights reserved 0007-0920/95 $12.00

### The novel fluorinated 2-nitroimidazole hypoxia probe SR-4554: reductive metabolism and semiquantitative localisation in human ovarian cancer multicellular spheroids as measured by electron energy loss spectroscopic analysis

EO Aboagye[1], AD Lewis[1], A Johnson [2], P Workman [1,3], M Tracy[4] and IM Huxham[2]

[1]*CRC Department of Medical Oncology, University of Glasgow, Beatson Laboratories, Switchback Road, Glasgow G61 1BD, UK;* [2]*Division of Molecular and Cellular Biology, University of Glasgow, Glasgow G12 8QQ, UK;* [3]*Present address: ZENECA Pharmaceuticals, Cancer Research Department, Macclesfield SK10 4TG, UK;* [4]*Life Sciences Division, SRI International, Menlo Park, California 94025, USA.*

**Summary** The novel fluorinated 2-nitroimidazole SR-4554 is undergoing preclinical development as a magnetic resonance spectroscopy and imaging probe for hypoxic tumour cells. We have used electron energy loss spectroscopic analysis (EELS) to show selective reduction and differential subcellular localisation of SR-4554 in human ovarian multicellular spheroids. SR-4554 was demonstrated to be metabolised by these A2780 cells under hypoxic but not under normal aerobic cell culture conditions. The EELS technique illustrated that the relative amount of drug within the cytoplasm of cells from both the inner region (150-160 μm from edge) and outer edge of the spheroid did not differ significantly after an initial 3 h incubation with drug. In contrast, an 8-fold differential between the amount of drug retained in the cytoplasm (primarily ribosomes and endoplasmic reticulum) of cells from the inner *vs* outer regions of the spheroids was observed following a subsequent 2 h 'chase' culture in drug-free medium. Within cells from the hypoxic region of the spheroid, SR-4554 was mainly associated with the endoplasmic reticulum, nucleus and the cytoplasmic side of intracellular vesicles and also to a lesser extent with the nuclear periphery. Interestingly, the drug was only weakly associated with the mitochondria and plasma membrane of the cells. The characteristics of cellular and subcellular distribution of SR-4554 are consistent with the hypothesis that 2-nitroimidazole compounds undergo hypoxia-mediated enzymatic reduction to reactive species. These reactive species are selectively retained in the cells in which they are metabolised through covalent association with subcellular components. These findings provide additional support for the clinical development of the drug as a non-invasive probe for tumour hypoxia and at the same time illustrate the utility of the EELS technique for examining the heterogeneity of drug distribution both between and within cells.

**Keywords:** electron energy loss spectroscopy; fluorinated 2-nitroimidazole; hypoxia probe

Human and animal tumours have been reported to contain regions of low oxygen tension or hypoxia (Thomlinson and Gray, 1955; Moulder and Rockwell, 1984; Rampling *et al.*, 1994). Tumour cells existing under hypoxia may be more resistant to therapy for a variety of reasons. These factors include: the requirement for molecular oxygen in the fixation of radiation-induced radicals and drug-induced damage, and certain drug activation processes; non-cycling cell kinetics; and decreased drug uptake (Workman, 1991). In recent years strategies have been developed to identify hypoxic cells within tumours, in order to facilitate the rational selection of appropriate therapeutic regimens. In this regard, both surgically invasive and non-invasive techniques such as autoradiography, immunohistochemistry, magnetic resonance spectroscopy (MRS) and single positron emission tomography (SPET) have been used to provide a clinically relevant approach to identifying these hypoxic cells within tumours (Chapman, 1984; Maxwell *et al.*, 1988; Koh *et al.*, 1991; Kwock *et al.*, 1992; Lord *et al.*, 1993; Hodgkiss *et al.*, 1994; Raleigh *et al.*, 1994).

Nitroimidazoles, including misonidazole, have been under evaluation as potential diagnostic probes for hypoxic cells. This is because of the specific reductive metabolism of the drugs to reactive metabolites which bind to macromolecules within hypoxic cells (Miller *et al.*, 1982; Chapman *et al.*, 1983). The characteristics of binding have been evaluated in various cell lines and tumours, although the exact nature of the metabolite is not known (Miller *et al.*, 1982; Chapman *et al.*, 1983). Interestingly, previous subcellular fractionation studies in EMT6 cells with [$^{14}$C]misonidazole have indicated that 77% and 23% of the total activity is associated with the acid-insoluble (bound) and the acid-soluble fractions (unbound) respectively (Miller *et al.*, 1982). Further, the acid-insoluble fraction is distributed among RNA (17%), DNA (1%), lipid (4%) and protein (1%) (Miller *et al.*, 1982). In another study by the Edmonton group, the involvement of intracellular enzymes in the activation process was implicated, since the temperature dependence of this process showed an activation energy of 33.5 kcal mol$^{-1}$ (Chapman *et al.*, 1983). This study also demonstrated that the binding rate of misonidazole within hypoxic cells was at least 50 times greater than within aerobic cells, and suggested that the activation sites may be lipid associated. The higher rate of binding and longer half-life of the bound metabolite compared with the parent unmetabolised compound are considered as favourable characteristics with regard to the use of 2-nitroimidazoles as possible markers for hypoxic cells.

Electron energy loss spectroscopic analysis (EELS) by electron spectroscopic imaging (ESI) is an emerging technique for the *in situ* examination of objects and compounds in cells and tissues, but has only recently been applied to an analysis of cancer therapeutic compounds (Huxham *et al.*, 1992, 1993). Briefly, the technique relies upon measurement of the electron energy distribution of transmitted electrons that have lost energy following interaction with a specimen. This is done by recording an energy spectrum as a sequence of images with high spatial resolution. These filtered electron image sequences contain information about the element-specific energy loss population (equivalent to energy loss edge) superimposed on the non-specific energy loss population (equivalent to background). Data are processed mathematically to obtain edge intensity information which relates directly to the elemental concentration of the specimen. Com-

Correspondence: AD Lewis
Received 18 January 1995; revised 9th March 1995; accepted 16 March 1995

paring the intensity of two elements, as performed in our study, can thus disclose semiquantitative elemental information on a local (nm) scale.

In the present investigation, we used energy electron loss spectroscopy to study the localisation of a novel fluorinated 2-nitroimidazole SR-4554 (Figure 1) within A2780 human ovarian multicellular spheroids. The A2780 spheroid model was chosen because it provides the coexistence of both aerobic and hypoxic cells under normal aerobic cell culture conditions, allowing manipulations on both types of cells to be carried out simultaneously. Moreover, we have previously used this spheroid model with EELS (Huxham *et al.*, 1992, 1993) to assess the localisation of fluorine-containing drug bound to macromolecules within different cell populations and subcellular compartments. SR-4554 was designed to contain three equivalent fluorines within a metabolically stable side-chain of appropriate lipophilicity, while retaining a similar reduction potential to misonidazole. Currently, SR-4554 is undergoing preclinical evaluation as a non-invasive probe for tumour hypoxia by fluorine-based magnetic resonance spectroscopy (MRS) and imaging (MRI). Together with bioreductive metabolism studies which are reported here, the present localisation studies will help in our understanding of the characteristics of metabolism-induced binding of SR-4554 and of 2-nitroimidazoles in general.

### Materials and methods

### *Metabolism studies*

SR-4554 [*N*-(2-hydroxy-3,3,3 trifluoropropyl)-2-(2-nitro-1-imidazolyl) acetamide] was synthesised and supplied by SRI International, Menlo Park, CA, USA. A2780 cells were cultured as monolayers in RPMI medium (Life Technology, Paisley UK) supplemented with 10% (v/v) fetal calf serum (Globepharm, Esher, Sussex, UK), and 0.001% (w/v) insulin (Lewes, Sussex, UK). The cells were grown to near confluence, trypsinised and plated at a concentration of $5 \times 10^6$ cells $ml^{-1}$ in 5 ml. Cells were allowed to adhere to the flasks and the medium replaced with one containing 10 and 20 μM SR-4554. The flasks were then incubated for various lengths of time up to 3 h under hypoxic (98% nitrogen and 2% carbon dioxide) or aerobic (2% carbon dioxide and 20% oxygen in nitrogen) conditions. Drug-containing media obtained at 0, 0.5, 1, 2, 3 h were then analysed by high-performance liquid chromatography (HPLC).

### *HPLC analysis and rate of SR-4554 reduction*

Aliquots (250 μl) of the incubation media were spiked with 20 μl of an internal standard (8 μg $ml^{-1}$ Ro 07-0269 [1-(2-nitro-1-limidazolyl)-3-chloro-2-propanol] supplied by Roche, Welwyn Garden City, Herts, UK) and the mixture extracted with 25 μl of silver nitrate solution (30%, w/v). Samples were vortexed, centrifuged (at 1000 *g*) for 10 min, and the supernatants analysed by HPLC (Millipore UK Ltd, Watford, UK) using a $C_{18}$ μBondapak analytical column and a mobile phase consisting of 15% methanol and water. The analytes were eluted at a flow rate of 2 ml $min^{-1}$, and the column effluent monitored at a wavelength of 324 nm. Calibration standards (0.35–35.46 μM) were prepared in RPMI medium and analysed under identical conditions to that above. The concentrations of parent drug in the incubation samples were determined and plotted against time. Rates of reduction were estimated from the initial slope of the concentration of SR-4554 *vs* time curve, which was linear within the time period studied.

**Figure 1** Drug structure of SR-4544.

### *Spheroid culture and EELs analysis*

Human ovarian A2780 cells were plated (in RPMI medium) at a concentration of $2 \times 10^6$ cells 50 $ml^{-1}$ and incubated in stirrer flasks at 37°C to form spheroids. After 3.5 days in culture, spheroids of approximately 0.8–1.4 mm in diameter were obtained for use in the EELS experiment. Initially the spheroids formed as aggregates, but after 3.5 days in culture they formed tight proliferating spheroids. Under these conditions the large spheroids develop a region of hypoxia between the outer cells and the necrotic core. For the present experiments, the outer most cell layer of the spheroid was taken to be aerobic, whereas cells in the inner region of the spheroid, at approximately 150–160 μm from the surface, but separate from any necrotic core, were selected as hypoxic as shown by Miller *et al.* (1989) using EMT6 spheroids. A2780 spheroids are similar to EMT6 spheroids in that both form tight spheroid structures. In this regard, spheroid models showing differences in oxygen levels and binding of 2-nitroimidazoles have previously been established (Franko *et al.*, 1987; Franko *et al.*, 1992).

The spheroids were incubated with culture medium containing a non-toxic concentration of 1 mM SR-4554 at 37°C for 3 h. Incubations were carried out under normal aerobic cell culture conditions. Half of the spheroids were then transferred onto ice to stop further reaction. The other half were washed with fresh culture medium and 'chased' at 37°C for 2 h under normal aerobic conditions. These were also transferred onto ice to stop further reaction.

For analytical electron microscopy, all spheroids were briefly washed in phosphate-buffered saline (PBS), and chemically fixed (on ice) with 1% glutaraldehyde in PBS for 1.5 h. The spheroids were dehydrated in a series of alcohols for embedding at low temperature in Lowicryl K4M, a nitrogen-free hydrophilic methacrylate resin, without the use of heavy metal stains to avoid electron scattering. Ultrathin sections were mounted onto 700 mesh copper grids for electron spectroscopic analysis using a Zeiss TEM902 microscope (Karl Zeiss Oberkochen, Oberkochen, Germany) operating at 80 kV and 12 000 × magnification. The microscope was fitted with an electron energy filter for analysis of the local electron energy associated with fluorine (K-edge onset at $\Delta E = 688$ eV) and nitrogen (K-edge onset at $\Delta E = 405$ eV). This novel technique has a resolution of about 2 nm and an energy resolution of about 5eV (Huxham *et al.*, 1992; Johnson *et al.*, 1995) for sections of resin-embedded cells, and a theoretical detection limit for fluorine of about 500 atoms.

Energy-filtered image sequences were recorded between $\Delta E = 650$ and 750 eV for fluorine and between $\Delta E = 350$ and 450 eV for nitrogen, changing only the (measurable) video-camera (Dage SIT, Michigan, MN, USA) kV setting between each sequence pair to accommodate the change in energy loss intensity relative to the dynamic range of the video-camera. Comparisons between defined regions within peripheral (outer) cells and cells approximately 150–160 μm from the edge of the spheroid (inner cells) were made using in-house software.

Energy loss contributions of both fluorine and nitrogen were measured following background modelling for each pixel in median filtered energy loss image sequence. This was accomplished using the least mean square determination of the parameters which describe the energy loss curve (deBruijn *et al.*, 1993; Johnson *et al.*, 1995). Cumulative background-stripped grey level values from 12 image sequences over a 30

eV portion of the post-ionisation edge for fluorine and nitrogen were used to calculate elemental ratios. In this way, variations in the density of cellular material between one domain and another could be normalised as a function of the nitrogen content for each region of interest, to produce local semiquantitative elemental maps. Data were also expressed as average grey level value representing energy loss for fluorine as a function of section area for each pixel.

### Results

### *Metabolism of SR-4554 by human ovarian carcinoma cells*

SR-4554 was reduced by A2780 cells in culture under hypoxic conditions. This was assessed by chromatographic analysis of loss of parent drug metabolised by the cells in culture. Figure 2 shows a typical chromatogram of a hypoxic compared with an aerobic incubation sample also containing the internal standard (Ro 07-0269). No metabolites were observed. The rates of reduction of SR-4554 by A2780 cells under aerobic and hypoxic culture conditions are shown in Table I. These data, obtained only at 37°C, demonstrated the selective reduction of SR-4554 under hypoxia in contrast to normal aerobic conditions. As expected, the reduction rates increased with increasing substrate concentration.

### *Localisation of SR-4554 within human ovarian carcinoma cells by EELS*

EELS analysis of spheroids incubated with SR-4554 enabled the localisation of the fluorine atoms in subcellular components as well as across different regions within the spheroid. Figure 3 shows an unstained, energy filtered image (reversed contrast) of A2780 cells from a spheroid after culture in SR-4554-containing medium, recorded at $\Delta E = 150$ eV. By means of spectroscopic imaging, most intracellular structures of unstained material, otherwise difficult to see without filtering, could be identified for analysis. Using our in-house image analysis software, regions of interest (ROI) for each electron spectroscopic image sequence (ESIS) were defined simply by drawing on the

**Figure 2** Typical HPLC plot of SR-4554 after incubation with A2780 cells for 30 min under (**a**) aerobic and (**b**) hypoxic conditions. The conditions of HPLC were as described in the Materials and methods section. From left to right the three peaks illustrated indicate the solvent front, SR-4554 and internal standard (IS; Ro 07-0269) respectively.

**Table I** Rate of loss of SR-4554 incubated with A2780 cells

| *Conditions* | *Drug concentration (μM)* | *Rate of loss (nmol h$^{-1}$ 10$^{-6}$ cells)* |
|---|---|---|
| Aerobic | 20 | No loss detected |
| | 10 | No loss detected |
| Hypoxic | 20 | 7.54 ± 0.10 |
| | 10 | 4.32 ± 0.25 |

SR-4554 was incubated with A2780 cells under aerobic and hypoxic conditions. The levels of parent drug remaining at various time periods were determined as described in the Materials and methods section. The values for rate of loss of compound are means ± s.d. from at least three separate determinations.

**Figure 3** Energy-filtered image of a portion of a heavy metal-free A2780 cell within SR-4554-treated multicellular spheroids after a 'chase' culture, recorded at $\Delta E = 150$eV, showing enhanced contrast in phosphorus-rich regions. The image shows a well-defined nuclear region (N), nuclear membrane (arrow), mitochondria (M) and a cytoplasmic region rich in polyribosomes (PR). Bar = 600 nm.

reference image on the display screen. The same ROI was used for analysis of both nitrogen and fluorine ESIS sequences. The energy losses for nitrogen and fluorine were then determined by plotting the cumulative grey level value for the ROI for each ESIS. A representative projected distribution of fluorine (green) within a section of the inner region of the spheroid after the 'chase' process is shown in Figure 4. This represents residual bound drug within the cell.

An analysis of cytoplasmic domains (which includes cytoplasm, mitochondria, vesicle periphery and plasma membrane regions) of cells within A2780 spheroids (Figure 5) suggest that the drug was distributed into cells across the whole spheroid after a 3 h culture. Following chemical fixation of these spheroids, only marginally less drug was bound to intracellular components within cells of the inner region of the spheroid as compared with the equivalent components of cells in the outer region. More importantly, an 8-fold higher level of drug was present within the cytoplasmic domains of these inner cells after 'chasing' with drug-free media, as most of the drug which was present in the outer cells had diffused

**Figure 4** (**a**) Reference image of a section from an inner A2780 cell within SR-4554-treated multicellular spheroids after a 'chase' culture, recorded $\Delta E = 150eV$. The image shows the plasma membrane (PM), vesicles (V), cytoplasm (C); nuclear membrane (NM), nucleus (N) and nucleolus (Nclo). (**b**) The same image upon which the projected fluorine distribution is superimposed (green). The green binary fluorine map simply shows regions in which fluorine was found to be present (i.e. a stripped grey level value above background). TIFF images were reproduced on a Kodak Colourease printer (field width = 5 $\mu$m).

away following the 'chase' process. This selective retention of drug by the inner cells is indicative of increased bioactivation within the inner cells of the spheroids.

Table II demonstrates that the drug distribution present within the cells from both the inner and outer regions of 'chased' spheroids was not uniform. Relative to the intrinsic nitrogen content of specific intracellular regions, which takes into account local variations in biological material, the amount of drug within the inner cells was found to be comparatively high at the periphery of intracellular vesicles, within the cytoplasm, in the nucleus and at its periphery. In contrast, the amount of drug was comparatively low within mitochondria and at the plasma membrane. Although the nitrogen atoms in the drug will contribute to the total nitrogen intensity signal of the EELS technique, by far the greater contribution will come from the biological macromolecules of the cell to which the drug is localised. When simply expressed in terms of the average amount of fluorine per unit area of cell section, the data suggested that there was a relative accumulation of fluorine at the nuclear periphery and in the nucleus compared with other regions, more representative of the projected fluorine distribution shown in Figure 4. In contrast, and as expected, across all domains there was less drug (3 to 10-fold) localised within the outer cells than in the inner cells.

**Figure 5** Histograms showing the mean ± s.d. of fluorine/nitrogen (F/N) elemental ratios for cytoplasmic domains (which includes cytoplasm, mitochondria, vesicle periphery and plasma membrane regions) from the outer and inner cells of A2780 multicellular spheroids cultured for 3 h (■) in SR-4554 and after a 2 h 'chase' culture (□) in drug-free media. Using the Mann-Whitney *U*-test (non-parametric), a significant difference was determined between the outer and inner cells after the 'chase' period ($P \leqslant = 0.05$).

### Discussion

The localisation of 2-nitroimidazoles following reduction by tumour cells is important in our understanding of the use of these compounds as hypoxic probes. The human ovarian A2780 cell line was used in this study since this cell line has previously been shown to express cytochrome P450 reductase, an enzyme involved in the reductive metabolism of 2-nitroimidazoles, by enzyme assay and PCR analysis (data not shown). This cell line was also considered likely to develop a hypoxic region in spheroids over the size range used.

The characteristics of the metabolism-induced binding of 2-nitroimidazoles to macromolecules in hypoxic tissue are still not fully understood. In particular, the nature of the drug adducts and the subcellular localisation of these compounds has been poorly addressed in the literature to date. This paper addresses some of these issues with respect to the use of a novel fluorinated 2-nitroimidazole (SR-4554) in A2780 cells/spheroids and the application of the EELS technique. However, these results are also relevant to the action of other 2-nitroimidazoles. The structure of SR-4554 is based on that of etanidazole (Brown and Workman, 1980), while its lipophilicity is more similar to misonidazole. The lipophilicities for SR-4554, etanidazole and misonidazole being 0.634, 0.046, and 0.430 respectively. Interestingly, the distribution properties demonstrated in our study are more appropriate in terms of those of etanidazole-like oxygen-dependent binding of the compound rather than misonidazole (Workman, 1982; Franko *et al.*, 1987; Kocha *et al.*, 1993). As expected, SR-4554 was metabolised (reduced) selectively by hypoxic A2780 cells but not under aerobic conditions.

The use of the novel EELS technique permitted the localisation of the compound within human ovarian carcinoma spheroids. In cultures not subjected to 'chasing', no significant differential was observed between cells of the inner and outer regions of the spheroid. This presumably is due to the presence of both original parent drug and bound drug metabolites. Since the EELS technique maps only atoms such as fluorine, nitrogen and phosphorus it cannot distinguish between original parent and bound drug. The method of 'chasing' in drug-free media to differentiate between bound and unbound drug was, therefore, employed to allow the assessment and localisation of bound metabolites. Interestingly, the results we obtained in our study using spheroids and EELS were in accordance with metabolism-induced binding of 2-nitroimidazoles to macromolecules as previously demonstrated by other methods with limited spatial resolution such as autoradiography and immunohistochemistry (Miller *et al.*, 1982; Lord *et al.*, 1993). In contrast to these other methods, the EELS technique also has the ability to measure drug binding relation to the density of other local macromolecular components. Although some non-specific binding occurred in the cells of the outer (aerobic) region of

**Table II** The relative average fluorine and fluorine/nitrogen elemental ratios for six intracellular domains of both outer and inner cells from A2780 multicellular spheroids following culture with SR-4554

| *Domain* | *Nucleus* | *Nuclear periphery* | *Cytoplasm* | *Mitochondria* | *Vesicle periphery* | *Plasma membrane* |
|---|---|---|---|---|---|---|
| *F/N ratio* | | | | | | |
| Outer | 0.03 ± 0.02[a] | 0.03 ± 0.01 | 0.11 ± 0.04 | ND[b] | 0.07 ± 0.03 | ND |
| Inner | 0.21 ± 0.02 | 0.25 ± 0.08 | 0.29 ± 0.08 | 0.06 ± 0.02 | 0.31 ± 0.06 | 0.16 ± 0.05 |
| *F/pixel* | | | | | | |
| Outer | 0.65 ± 0.28 | 0.45 ± 0.19 | 0.75 ± 0.22 | ND | 0.61 ± 0.18 | ND |
| Inner | 2.15 ± 0.55 | 4.25 ± 0.75 | 1.97 ± 0.46 | 0.85 ± 0.22 | 0.25 ± 0.06 | 0.49 ± 0.13 |

[a]Average value from 12 areas ± s.d. for each domain expressed as the relative amount of fluorine per pixel (8 $nm^2$) (F/pixel) or as a fluorine to nitrogen ratio (F/N ratio) in arbitary units.
[b]ND, not detected. Mann-Whitney *U*-test 5% significance: for *F/N ratio*: *outer cells*, cytoplasm *vs* nuclear periphery and cytoplasm *vs* nucleus; *inner cells*, vesicle periphery *vs* mitochondria and vesicle periphery *vs* plasma membrane, mitochondria *vs* nucleus, mitochondria *vs* nuclear periphery and mitochondria *vs* cytoplasm. For *F/pixel*: *outer cells*, no significant differences observed; *inner cells*, mitochondria *vs* nucleus and mitochondria *vs* nuclear periphery, nuclear periphery *vs* cytoplasm, nuclear periphery *vs* vesicle periphery and nuclear periphery *vs* plasma membrane.

the spheroids, levels of fluorine in the cytoplasm of cells from the inner (hypoxic) regions were 8-fold higher. This effect is the more likely to occur *in vivo*, where elimination processes will result in the removal of unbound drug compared with the bound metabolite. Importantly, the localisation characteristics shown in our study are very relevant to the use of this novel fluorinated compound as a non-invasive marker of tumour hypoxia in the clinic. The presence of three magnetically equivalent fluorine atoms in the structure of the compound, which remains intact on enzyme reduction, is important in terms of its detection by resonance techniques as well as by EELS.

Further studies using EELS enabled us to analyse in more detail the subcellular localisation of the compound within cells of both the outer and inner region of the spheroid. In our study, the mitochondria and plasma membrane did not appear to bind significant amounts of drug. Of interest, however, were the high levels of the compound localised to the nuclear periphery, nucleus and cytoplasm within the inner cells. These data are in agreement with previous published studies using subcellular fractionation of cells labelled with [$^{14}$C]misonidazole (Miller *et al.*, 1982), considering that the molecular components in these regions consist mainly of RNA, DNA, lipids and proteins. In addition, the distribution of 2-nitroimidazole to cytoplasm, nuclear and perinuclear regions was also mentioned by Cline *et al.* (1994), who used antibodies against CCI-103F/CCI-103F adducts to follow the distribution of hypoxia and/or reductive enzymes within cells of canine tumours. In contrast, and as expected, less drug was distributed within all the domains in the outer cells.

The initial steps in the reductive metabolism of 2-nitroimidazoles are mainly catalysed by cytochrome P450 reductase and to a lesser extent cytochrome P450, both of which are found in the endoplasmic reticulum (McManus *et al.*, 1982; Walton and Workman, 1987). The subcellular distribution of the SR-4554 compound demonstrated in our study suggests that the reactive intermediate is short-lived and binds to macromolecules within the vicinity of the metabolism site or to nucleophiles such as RNA close to these sites. Importantly, the evidence would suggest that the reactive metabolites do not appear to migrate out of the cells. This characteristic is also relevant to the design of bioreductive drugs, based on 2-nitroimidazoles, which will target the nucleus of the cell to deliver radioisotopes or alkylating moieties.

Currently, SR-4554 is undergoing preclinical development before scheduled clinical trials as a probe for investigating tumour hypoxia by non-invasive MRS. Important to the direction of these studies, this paper describes the intracellular distribution of the compound within hypoxic regions of human ovarian carcinoma spheroids and is useful in the interpretation of data from MRS studies. In addition, however, the EELS technique itself can also be used to study the distribution of hypoxic regions within tumours labelled *in vivo* with SR-4554, even though this will involve invasive (biopsy) procedures. As a semiquantitative technique, moreover, it offers the potential of measuring hypoxia on a cell-to-cell basis at a molecular level with good resolution compared with antibody techniques and also the investigation of drug to macromolecule interactions without the use of radioisotopes.

#### Acknowledgements

The authors would like to thank Peter McHardy for his assistance in the preparation of graphic material, and also the financial support of the Cancer Research Campaign (UK), Overseas Research Scholarship (awarded to EOA) and Scottish Hospitals Endowment Research Trust. PW acknowledges the award of a CRC Life Fellowship.

#### References

BROWN JM AND WORKMAN P. (1980). Partition coefficient as a guide to the development of radiosensitizers which are less toxic than misonidazole. *Radiat. Res.*, **82**, 171–190.

CHAPMAN JD. (1984). The detection and measurement of hypoxic cells in solid tumours. *Cancer*, **54**, 2441–2449.

CHAPMAN JD, BAER K AND LEE J. (1983). Characteristics of the metabolism-induced binding of misonidazole to hypoxic mammalian cells. *Cancer Res.*, **43**, 1523–1528.

CLINE JM, THRALL DE, ROSNER GL AND RALEIGH JA. (1994). Distribution of the hypoxia marker CCI-103F in canine tumours. *Int. J. Radiat. Oncol. Biol. Phys.*, **28**, 921–933.

DE BRUIJN W, SORBERS C, GELSEMA E, BECKERS A AND JONKIND J. (1993). Energy filtering transmission electron microscopy of biological specimens. *Scanning Microsc.* **7**, 693–709.

FRANKO AJ, KOCH CJ, GARRECHT BM, SHARPLIN J AND HUGHES D. (1987). Oxygen dependence of binding of misonidazole to rodent and human tumours *in vitro*. *Cancer Res.*, **47**, 5367–5376.

FRANKO AJ, KOCH CJ AND BOISVERT DP. (1992). Distribution of misonidazole adducts in 9L gliosarcoma tumors and spheroids: implications for oxygen distribution. *Cancer Res.*, **52**, 3831–3837.

HODGKISS RJ, PARRICK J, PORSSA M AND STRATFORD MRL. (1994). Bioreductive markers for hypoxic cells: 2-nitroimidazoles with biotinylated substituents. *J. Med. Chem.*, **37**, 4352–4356.

HUXHAM IM, GAZE MN, WORKMAN P AND MAIRS RJ. (1992). The use of parallel EEL spectral imaging and elemental mapping in the rapid assessment of anti-cancer drug localization. *J. Microsc.*, **166**, 367–380.

HUXHAM IM, BARLOW A, MAIRS R, GAZE M AND WORKMAN P. (1993). Elemental mapping of fluorine using ESI for the localisation of an anthracycline drug ME2303 in human ovarian carcinoma cells. *Cell Biol. Int.*, **17**, 685.

JOHNSON AD, MAIRS RJ, GAZE MN, SASS G AND HUXHAM IM. (1995). Electron spectroscopic imaging of organic compounds using PC-based energy sequence imaging software. *Microsc. Micronal. Microstruct.*, **6**, 1–13.

KOCH CJ, GIANDOMENICO AR AND IYENGAR CWL. (1993). Bioreductive metabolism of AF-2 [2(2-furyl)-3-(5-nitro-2-furyl)acrylamide] combined with 2-nitroimidazoles: implication for use as hypoxic cell markers. *Biochem. Pharmacol.*, **46**, 1029–1036.

KOH WJ, RASEY JS, EVANS ML, GRIERSON JR, LEWELLEN TL, GRAHAM MM, KROHN KA AND GRIFFIN TW. (1991). Imaging of hypoxia in human tumours with [F-18]fluoromisonidazole. *Int. J. Radiat. Oncol. Biol. Phys.*, **22**, 199–212.

KWOCK L, GILL M, MCMURRY HL, BECKMAN W, RALEIGH JA AND JOSEPH AP. (1992). Evaluation of a Fluorinated 2-nitroimidazole binding to hypoxic cells in tumour-bearing rats by $^{19}$F magnetic resonance spectroscopy and immunohistochemistry. *Radiat. Res.*, **129**, 71–78.

LORD EM, HARWELL L AND KOCH CJ. (1993). Detection of hypoxic cells by monoclonal antibody recognizing 2-nitroimidazole adducts. *Cancer Res.*, **53**, 5721–5726.

MCMANUS ME, LANG MA, STUART K AND STRONG J. (1982). Activation of misonidazole by rat liver microsomes and purified NADPH-cytochrome C reductase. *Biochem. Pharmacol.*, **31**, 547–552.

MAXWELL RJ, WORKMAN P AND GRIFFITHS JR. (1988). Demonstration of tumour-selective retention of fluorinated probe by $^{19}$F magnetic resonance spectroscopy *in vivo*. *Int. J. Radiat. Oncol. Biol. Phys.*, **16**, 925–929.

MILLER GG, NGAN-LEE J AND CHAPMAN JD. (1982). Intracellular localization of radioactively labelled misonidazole in EMT-6 tumour cells *in vitro*. *Int J. Radiat. Oncol. Biol. Phys.*, **8**, 741–744.

MILLER GG, BEST MW, FRANKO AJ, KOCH CJ AND RALEIGH JA. (1989). Quantitation of hypoxia in multicellular spheroids by video image analysis. *Int. J. Radiat. Oncol. Biol. Phys.*, **16**, 949–952.

MOULDER JE AND ROCKWELL S. (1984). Hypoxic fractions of solid tumours: Experimental techniques, methods of analysis, and a survey of existing data. *Int. J. Radiat. Oncol. Biol. Phys.*, **10**, 695–712.

RALEIGH JA, LA DINE JK, CLINE JM AND THRALL DE. (1994). An enzyme-linked immunosorbent assay for hypoxia marker binding in tumours. *Br. J. Cancer*, **69**, 66–71.

RAMPLING R, CRUICKSHANK G, LEWIS AD, FITZSIMMONS S AND WORKMAN P. (1994). Direct measurement of $pO_2$ distribution and bioreductive enzymes in human malignant brain tumours. *Int. J. Radiat. Oncol. Biol. Phys.*, **29**, 427–431.

THOMLINSON RH AND GRAY LH. (1955). The histological structure of some human lung cancers and the possible implications for radiotherapy. *Br. J. Cancer*, **9**, 539–549.

WALTON MI AND WORKMAN P. (1987). Nitroimidazole bioreductive metabolism: quantitation and characterisation of mouse tissue benznidazole nitroreductases *in vivo* and *in vitro*. *Biochem. Pharmacol.*, **36**, 887–896.

WORKMAN P. (1982). Lipophilicity and the pharmacokinetics of nitroimidazoles. In *Advanced Topics on Radiosensitizers of Hypoxic Cells*, Breccia A, Rimondi C and Adams GE. (eds) pp. 143–163. Plenum Press: Amsterdam.

WORKMAN P. (1991). Keynote address: bioreductive mechanisms. *Int. J. Radiat. Oncol. Biol. Phys.*, **22**, 631–637.